# A Comparison of Different Tissues Identifies the Main Precursors of Volatile Substances in Chicken Meat

**DOI:** 10.3389/fphys.2022.927618

**Published:** 2022-07-07

**Authors:** Na Luo, Li Liu, Xiaoya Yuan, Yuxi Jin, Guiping Zhao, Jie Wen, Huanxian Cui

**Affiliations:** State Key Laboratory of Animal Nutrition, Institute of Animal Sciences, Chinese Academy of Agricultural Sciences, Beijing, China

**Keywords:** chicken, meat, aroma precursor, breast muscle, abdominal fat

## Abstract

Amino acids and fatty acids are the main precursors of volatile organic compounds (VOCs) in meat. The purpose of this study was to determine the main VOC components in chicken breast muscle (BM) and abdominal fat (AF) tissue, as well as the source of VOCs, to provide a basis for quality improvement of broilers. BM and AF served as experimental and control groups, and gas chromatography-mass spectrometry (GC-MS) and untargeted metabolomics were employed to identify the source of VOCs. The results revealed nine VOCs in BM and AF tissues, including hexanal, octanal, and nonanal. VOCs including 1-octen-3-ol, (E,E)-2, 4-nonadienal, and benzaldehyde were significantly elevated in BM compared with AF (*p* < 0.05), while heptane and diethyl disulphide showed the opposite trend (*p* < 0.05). Levels of hexanal, heptanal, and octanal were similar in the two tissues. Metabolites of VOCs in chicken BM were investigated by weighted co-expression network analysis. However, only blue module in BM tissue was positively correlated with hexanal (*r* = 0.66, *p* = 0.01), heptanal (*r* = 0.67, *p* = 0.008), and (E,E)-2,4-nonadienal (*r* = 0.88, *p* = 3E-05). L-tyrosine, L-asparagine, adenosine, and valine were the main precursors of (E,E)-2,4-nonadienal and heptanal in BM tissue. Amino acids are the main precursors of 1-octen-3-ol, (E,E)-2, 4-nonadienal, and heptanal in chicken meat, while fatty acids are the main precursors of diethyl disulfide. However, hexanal can be synthesized from amino acids and small amounts of fatty acids as precursors. These findings expand our understanding of VOCs in chicken.

## Introduction

Meat products can be essential components of a balanced diet. Flavor is an essential sensory characteristic of the overall acceptability of meat and meat products including poultry. Flavor enjoyment is highly correlated with the whole experience of meat products, and consumers reject products with an unpleasant flavor (Sitz et al., 2005). Therefore, improving the flavor quality of broiler chicken is important to appeal to consumers.

Volatile organic compounds (VOCs) significantly influence the sensory characteristics of livestock and poultry meat. Over 600 VOCs have been identified in meat and meat products including poultry, such as hydrocarbons, aldehydes, ketones, esters, alcohols, furans, thiophenes, thiazoles, pyridines, and pyrroles (Wettasinghe et al., 2001; Feng et al., 2018). The thermally induced Maillard reaction and lipid oxidation contribute to the formation of the flavor (He et al., 2021). Fatty acids (FAs) and amino acids (AAs) are important precursors that affect the flavor of meat, and FAs in meat are related to the unique flavors of different meats (Mottram, 1998). The most crucial step in creating meat flavor is the Maillard reaction. Maillard reactions are a complex group of chemical reactions between carbonyl compounds (reducing sugars) and amino compounds (AAs and proteins). These reactions trigger many processes that lead to the formation of VOCs, as well as the characteristic brown color formation of cooked meat (Diez-Simon et al., 2019). Especially to flavor formation, the strecker degradation is of utmost importance. For flavor formation, Strecker degradation is of utmost importance. AAs are degraded *via* dicarbonyls formed in the Maillard reaction, leading to deamination and decarboxylation of AAs (Van Boekel, 2006). Both pleasant and unpleasant aroma compounds are produced from the catabolism of AAs (Urbach, 1993). AAs provide key nutritional value and contribute to the taste and flavor of meat (Ma et al., 2020).

Fat is the major contributor to flavor development in meat. Fatty tissues endow meat with specific flavor attributes, and this also depends on different kinds of FAs. Changes in FA composition can also affect flavor of meat (Aurousseau et al., 2004). Lipids are the sources of VOCs and are responsible for specific flavors. There are more unsaturated FA changes in FA deposition of ruminants and non-ruminants (Calkins and Hodgen, 2007). FAs have also been studied as flavor precursors in oxidized butter flavor (Song et al., 2022). FAs are the most critical contributors to flavor formation during meat cooking (Khan et al., 2015; Xiao et al., 2021), and fat produces flavors as it melts (Poleti et al., 2018). Linoleic and arachidonic FAs auto-oxidize and form 2-nonenal, 2,4-decadienal, 1-octen-3-ol, (E,E)-2,4-nonadienal, 2-octenal *via* 9-hydroperoxide and 11-hydroperoxide intermediates. Both 2-nonenal and 2,4-decadienal contribute a meaty flavor. Upon oxidation of arachidonic acid, the most intense aroma compound is trans-4,5-epoxy-(E)-2-decenal, followed by 1-octen-3-ol, 2,4-decadienal, 2,4,7-tridecatrienal, and hexanal. Polyunsaturated FAs (PUFAs) are rapidly heated and oxidized, producing VOCs components such as 2,4-decadienoal that can improve the flavor of meat (Calkins and Hodgen, 2007). Therefore, FAs and AAs are essential precursors of meat flavor.

Gas chromatography-mass spectrometry (GC-MS) is one of the main techniques employed for the analysis of VOCs (Creaser et al., 2004; Sun et al., 2020; Wang et al., 2020), including measuring their abundance (Chen et al., 2006; Mayr et al., 2015; Hopfer et al., 2016; Qi et al., 2018; Wang et al., 2019). Metabolomics has been applied to chickens to detect and analyze metabolites in cells, tissues, and organs. It has been used to explore differences in metabolites in BM from different chicken breeds (Tan et al., 2021; Zhang et al., 2022). AAs and FAs are the main precursors of VOCs in meat. However, the precursors of VOCs in chicken have not been confirmed. Wenchang chicken (WC), which is a local variety with high-quality meat flavor, was selected for the determination and comparison of VOCs in BM tissue, mainly composed of AAs and AF tissue, mainly composed of FAs, to explore the source of VOCs.

## Materials and Methods

### Animals and Sample Collection

WC chickens with the same genetic background were used in experiments at the Institute of Poultry Research (CAAS, Yangzhou, China) and reared in an environmentally controlled room with three-tier stepped cages from 1 day. Basal diets were formulated according to the requirements of the National Resources Committee (1994) and the Standards for Chicken Feeding (2004) developed by the Ministry of Agriculture, Beijing, China. At 98 days of age, 15 female WC chickens with similar body weight were stunned by electric shock and killed by bloodletting with a 12 h fast. After slaughter, BM and AF tissues were collected and stored at −80°C until use.

### Electronic-Nose Analysis

After thawing at 4°C, five BM and AF samples (6 g) were placed in closed sample bottles and heated at 100°C for 30 min. The Heracles II E-nose (Alpha MOS, Toulouse, France) were employed. This was based on rapid gas chromatography (GC) with two capillary columns of different polarity, a non-polar MTX-5 (5% diphenyl) and a medium polarity MXT1701 (14% cyanopropyl-phenyl) with dimensions of 10 m × 0.18 mm × 0.4 μm, and two ultrasensitive flame ionization detectors (μ-FIDs). The E-nose was also equipped with an Odor Scanner HS 100 autosampler (Gerstel GmbH, Mülheim, Germany). Each sample was incubated for 20 min at 40°C with agitation at 500 rpm. After incubation, only the headspace phase (2,500 μl) was transferred into the injector and heated to 200°C. The chromatographic parameters were an initial temperature of 70°C (maintained for 18 s), increased to 270°C at 2°C/s (maintained for 30 s), a detector temperature of 270°C and hydrogen N5.0 as a carrier gas (Linde Gaz, Krakow, Poland).

### Gas Chromatography-Mass Spectrometry Analysis

Pretreatment of each of 15 BM and AF samples was the same as for E-nose analysis. After thawing at 4°C, the 15 BM and AF samples (6 g) were placed in closed sample bottles and heated at 100°C for 30 min. Following headspace extraction, solid- phase microextraction (SPME) arrows were directly transferred and desorbed in the injection port of the GC for 5 min on splitless mode. Analytes were separated and detected using a split/splitless injector and a TC-5MS (30 m-0.25 mm-0.25 μm) fused silica column (Sigma-Aldrich, Saint Louis, MO, United States) using a helium flow rate of 1 ml/min. The column temperature was initially held at 40°C for 1 min, gradually increased to 280°C at a rate of 6°C min, then held at 280°C for 2 min. The MS instrument parameters were electron ionization (EI) 70 eV, ion source temperature 300°C and interface temperature 280°C. Full scan mode was used for MS detection at a mass range of 40–550 m/z. Each mass spectrogram corresponding to each chromatographic peak was qualitatively determined based on a computer chart. The relative content of each component was calculated by the peak area normalization method according to its total ion current chart.

### Metabolome Profiling

Metabolome profiling was carried out using a widely untargeted metabolome method by Novogene Co., Ltd. (Beijing, China; https://cn.novogene.com/). In brief, each of the 15 BM and AF samples were weighed, and 100 mg of each sample was ground using liquid nitrogen and placed in an Eppendorf tube with 500 μl of 80% aqueous methanol, vortex-shocked, left to stand for 5 min in an ice bath, and centrifuged for 20 min at 15,000 g and 4°C. An appropriate amount of supernatant was diluted with mass spectrometry grade water to 53% methanol. The supernatant was collected by centrifugation at 15,000 g and 4°C for 20 min and injected into the LC-MS instrument (Thermo Fisher Scientific Inc., Massachusetts, CA, United States) for analysis (Want et al., 2010). Quality control (QC) samples were taken from each experimental sample in equal volume and mixed as QC samples. Blank samples were 53% methanol aqueous solution instead of experimental samples, and the pretreatment procedure was the same as for experimental samples. A HypesilGoldcolumn (C18) column used was at a temperature of 40°C and a flow rate of 0.2 ml/min. Downstream data (.raw) files were imported into CD3.1 search library software for processing. Simple screening of parameters such as retention time and the mass-to-charge ratio was performed for each metabolite. Retention time deviation of 0.2 min and mass deviation of 5 ppm for peak alignment of different samples were employed to make the identification more accurate, followed by setting a mass deviation of 5 ppm, signal intensity deviation of 30%, the signal-to-noise ratio of 3, minimum signal intensity, summation ions and other information for peak extraction. Additionally, peak area quantification and integration of target ions were applied, followed by molecular ion peak and fragment ion for molecular formula prediction and comparison with mzCloud (https://www.mzcloud.org/), mzVault and Masslist databases. Background ions were removed using blank samples and normalizing the raw quantitative results, resulting in the identification and relative quantitation of metabolites.

### Weighted Gene Co-Expression Network Analysis

All individuals were employed to establish an unsigned co-expression network. The Weighted Gene Co-Expression Network Analysis (WGCNA) package (Langfelder and Horvath, 2008) was used to perform a weighted metabolite co-expression analysis with default settings, with minor modifications. Based on the WGCNA analysis of BM tissue (*n* = 15), we set the minModuleSize to 30 and the parameter of MEDissThres to 0.25 (soft threshold = 9). Metabolite module co-expression clustering dendrograms were built using the step-by-step topology overlap matrix (TOM), module detection, and similar module merging functions. Module-traits associations were quantified, and the Benjamin and Hochberg method (Benjamini and Hochberg, 1995) was used to adjust corresponding correlations.

### Analysis of Differential Metabolites

Fifteen BM and AF samples were used for differential analysis. Using multivariate statistical analysis, data were transformed using the metabolomics data processing software metaX (Wen et al., 2017) and subjected to principal component analysis (PCA) and partial least squares discriminant analysis (PLS-DA) to obtain variable importance in the projection (VIP) values for each metabolite. VIP is the variable projection importance of the first principal component (PC1) of the PLS-DA model (Heischmann et al., 2016). Using univariate analysis, *t*-tests were applied to calculate the statistical significance (*p*-value) of each metabolite between the two groups (Rao et al., 2016) and to calculate the fold change (FC) of metabolites between the two groups. Thresholds were set for VIP > 1.0, FC > 1.5 or FC < 0.667, and *p* < 0.05 (Sreekumar et al., 2009; Haspel et al., 2014).

### Pathway Enrichment Analyses

Kyoto Encyclopedia of Genes and Genomes (KEGG) pathway enrichment analysis was performed by Metaboanalyst 5.0 (https://www.metaboanalyst.ca/). The significance level was set at *p* < 0.05 for KEGG pathways.

### Statistical Analysis

VOCs were analyzed using Microsoft Excel 2019 (Microsoft Corporation, Redmond, WA, United States). Student’s t-test was employed to assess the significance of differences between groups using SPSS 22.0 (IBM Corp, Armonk, NY, United States). Confidence limits were set at 95% and *p < 0.05 or **p < 0.01 was considered significant. Results are presented as mean ± standard error of the mean (SEM). Plots were drawn using R software (version 4.1) and Graphpad prism 9 (GraphPad Software Inc.).

## Results

### Comparison of Volatile Organic Compounds in Breast Muscle and Abdominal Fat Tissues

**FIGURE 1:**
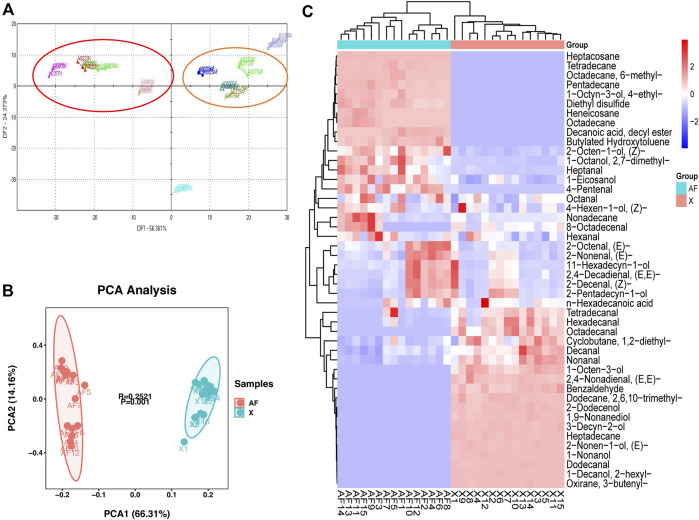
**(A)** Discriminant factor analysis (DFA) of E-nose results for different tissues in WC chickens. **(B)** PCA of BM and AF individuals. **(C)** Cluster diagram of volatile compounds.

Based on data from E-nose experiments, discriminant factor analysis (DFA) could effectively distinguish chicken BM and AF tissue of VOCs (Figure 1A). This study detected 36 and 32 VOCs in BM and AF tissues, respectively (Supplementary Table S1). The PCA results of all samples are shown in Figure 1B. Based on the PCA results, VOCs of BM and AF tissues could be distinguished, proving that the flavor quality of the two tissues differed. These results also showed that the accumulation of VOCs in different tissues was specific.

To explore the relationships between samples and differences in VOCs levels, all VOCs were hierarchically clustered. The content patterns of all VOCs in tissues were divided into three groups (Figure 1C). Some VOCs were lower in BM than in AF, including heptacosane, diethyl disulfide, heneicosane, octadecane, and other VOCs. Meanwhile, some VOCs were more abundant in BM than AF, including hexadecanal, octadecanal, nonanal, 1-octen-3-ol, (E,E)-2,4-nonadienal, benzaldehyde, heptadecane, 1-nonanol, dodecanal and other VOCs. Finally, levels of some VOCs were similar in the two tissues, such as hexanal, heptanal, 1-eicosanol, 4-pentenal, octanal, nonadecane, 8-octadecenal, (E)-2-nonenal, (E,E)-2,4-decadienal, and (Z)-2-decenal.

### Identification of Major Volatile Organic Compounds in Breast Muscle and Abdominal Fat Tissue

**FIGURE 2:**
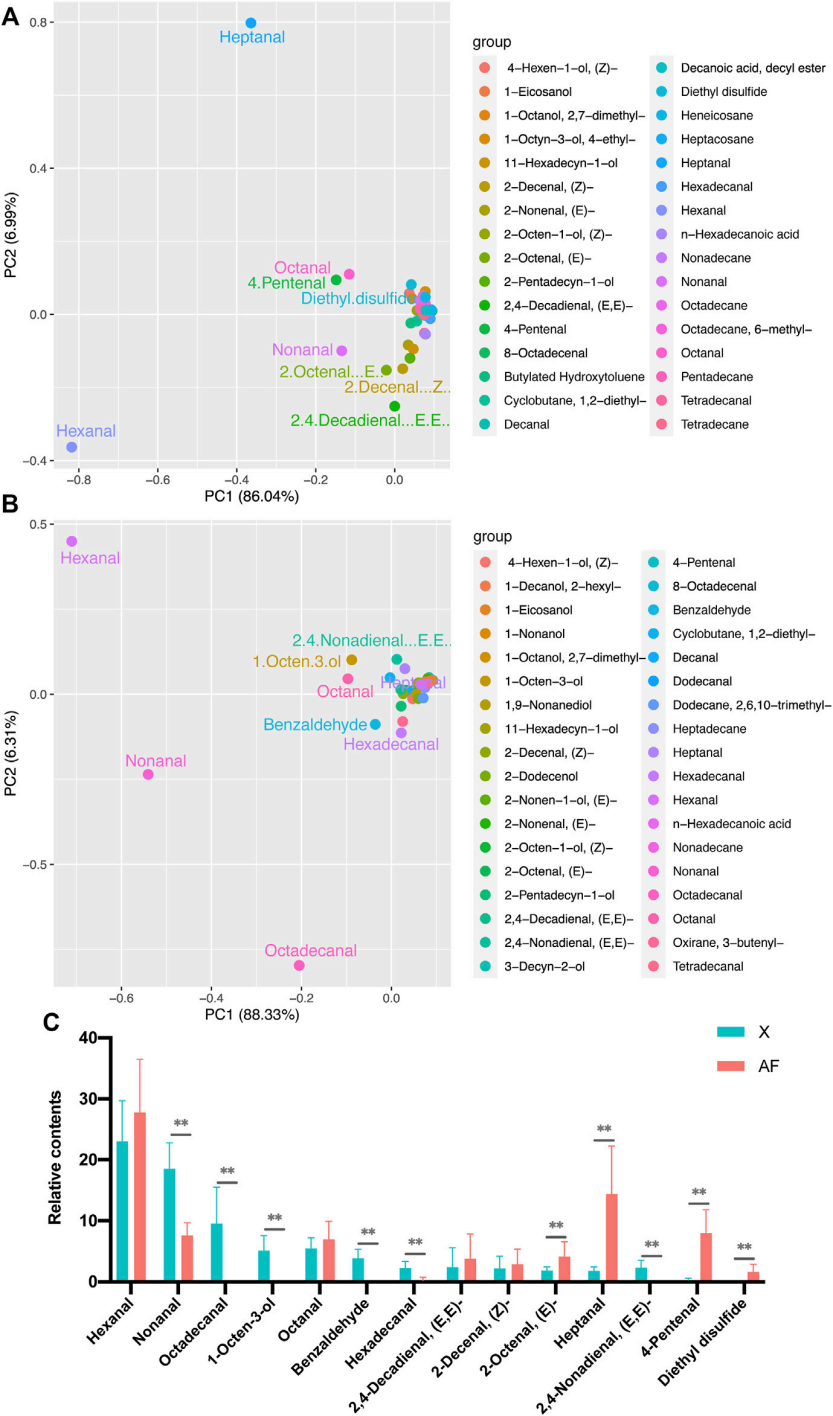
**(A)** PCA results of major volatile compounds in BM tissue. **(B)** PCA results for major volatile compounds in AF tissue. **(C)** Levels of major volatile compounds.

As shown in Figure 2, PCA results were obtained for all VOCs in the two tissues. BM contained nine major VOCs, including hexanal, heptanal, octanal, nonanal, octadecanal, benzaldehyde, (E,E)-2,4-nonadienal, hexadecanal, and 1-octen-3-ol. AF tissue also contained nine major VOCs, including hexanal, heptanal, octanal, nonanal, 4-pentenal, (Z)-2-decanal, (E)-2-octenal, (E,E)-2-decadienal, and diethyl disulfide. The main VOCs in both BM and AF tissue have four same VOCs, including hexanal, heptanal, octanal and nonanal.

Hexanal, 1-octen-3-ol and octanal contribute negatively to PC1 and positively to PC2 in BM. Nonanal, benzaldehyde and octadecanal contribute negatively to PC1 in BM and PC2. (E,E)-2,4-nonadienal and heptanal contribute positively to PC1 and PC2 in BM. Hexadecanal contributes positively to PC1 and negatively to PC2 in BM. PC1 explains 88.33% and PC2 explains 6.31% of the variation (Figure 2A). Heptanal, octanal and 4-pentenal contribute positively to PC1 in AF, and negatively to PC2. Hexanal, nonanal and (E)-2-octenal contribute negatively to PC1 in AF and PC2. (Z)-2-decenal and (E, E)-2,4-decadienal contribute positively to PC1 in AF and negatively to PC2. Diethyl disulfide contributes positively to PC1 in AF and positively to PC2. PC1 explains 86.04% of the variation and PC2 explains 6.99% (Figure 2B).

Comparing the relative content of the main VOCs in the two tissues, hexanal was the most abundant aldehyde, with levels higher in AF tissue than in BM tissue. Octadecanal, benzaldehyde, (E,E)-2,4-nonadienal, hexadecanal and nonanal were significantly higher in BM than in AF. Conversely, 4-pentenal, heptanal, hexanal, (Z)-2-decenal, (E,E)-2,4-decadienal, (E)-2-octenal and octanal were significantly lower in BM than in AF. 1-octen-3-ol, (E,E)-2,4-nonadienal, benzaldehyde and octadecanal were detected in BM but not in AF, while diethyl disulfide was detected only in AF (Figure 2C).

### Detection of Metabolites in Breast Muscle and Abdominal Fat Tissue

**FIGURE 3:**
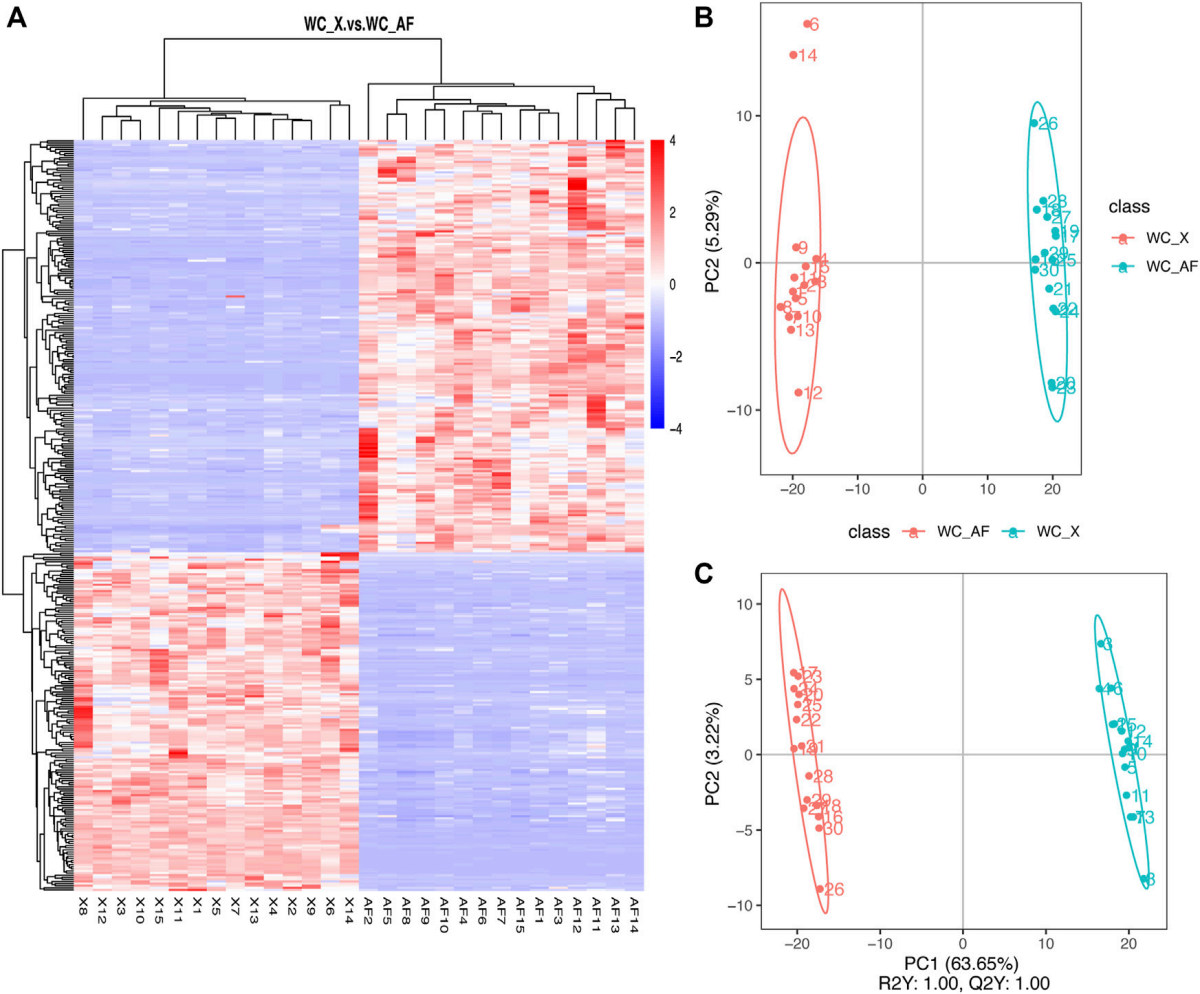
**(A)** Cluster diagram of all metabolites. **(B)** PCA of total samples. **(C)** PLS-DA of total samples.

Metabolites were detected in BM and AF tissue, and 620 metabolites were detected in BM tissue, compared with 682 metabolites in AF tissue. The same metabolites were hierarchically clustered to show the differences more intuitively in metabolite levels between samples (Figure 3A). We found that the metabolite accumulation patterns in different tissues of WC chickens were different. PCA was then performed on the metabolic profiles of 30 samples (2 × 15 biological replicates). The results showed that PC1 could explain 63.65% of the total variance of different tissues (Figure 3B). In comparison, PC2 could explain 5.29% of the total variance, and revealed significant difference in metabolite expression patterns between the two tissues. PLS-DA was also performed on BM and AF. Model evaluation parameters can be obtained by establishing a PLS-DA model of BM compared with AF (R2, Q2). The results show that R2 and Q2 are equal to 1, which indicates that the model is accurate and reliable (Figure 3C).

### Screening of Important Metabolites Related to the Scent of Meat

**FIGURE 4:**
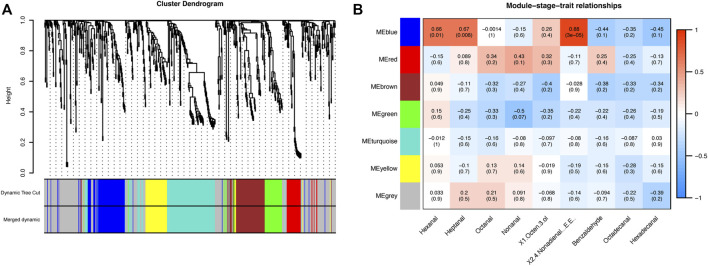
**(A)** Hierarchical clustering presenting seven modules with co-expressed metabolites in BM tissue. Each leaflet in the tree corresponds to individual metabolic pathways. **(B)** Module-stage-tissue relationships between metabolite modules and major volatile compounds in BM tissue. The upper number in the module is the correlation coefficient between modules and bottom traits, and the lower number is the *p*-value of the correlation coefficient.

To gain further insight into regulating metabolic changes in BM, WGCNA was performed to investigate the co-expression networks of 620 metabolites in BM tissue. We set MEDissThres as 0.25 to merge similar modules. Seven modules were generated in BM tissue (Figure 4A; Supplementary Table S2), but only the blue module was positively correlated with hexanal (*r* = 0.66, *p* = 0.01), heptanal (*r* = 0.67, *p* = 0.008), (E,E)-2,4-nonadienal (*r* = 0.88, *p* = 3E-05). The blue module included 82 metabolites (Figure 4B). The blue module mainly included D-Glutamine, Methionine, L-Aspartic acid, L-Serine, Valine, Dihydroxyacetone Phosphate and other metabolites.

**FIGURE 5:**
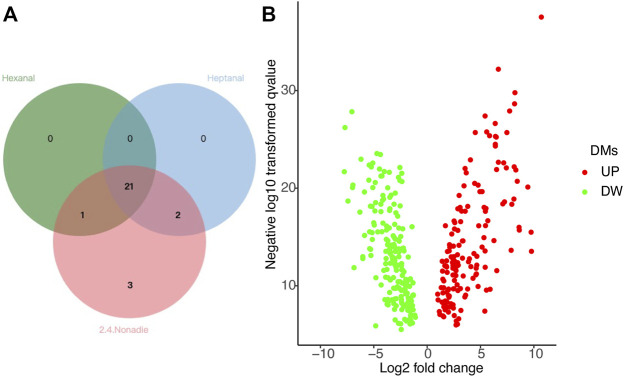
**(A)** Common DMs related to hexanal, heptanal, and (E,E)-2, 4-nonadienal, in the blue module of the BM tissue network. **(B)** Volcano plot of differential metabolites (DMs).

Next, we screen key metabolites in the blue module based on gene significance (GS) > 0.2, p.GS < 0.05, and module membership (MM) > 0.8. Based on hexanal, heptanal, and (E,E)-2,4-nonadienal traits in the blue module of BM tissue, we screened 22, 23, and 27 key metabolites, respectively. We eventually selected 21 common metabolites in BM tissue, including 6-Methylquinoline, L-Tyrosine, L-Asparagine, Adenosine, Valine and other metabolites (Figure 5A).

### Differential Metabolites in Breast Muscle Tissue

Differential Metabolites (DMs) related to heptanal and (E,E)-2,4-nonadienal between BM and AF were analyzed. Under the criteria VIP > 1.0, FC > 1.5 or FC < 0.667, and *p* < 0.05, there were 357 metabolites (Supplementary Table S3), including 161 upregulated and 196 downregulated in BM compared with AF (Figure 5B). By comparing DMs with significant correlation, 13 common metabolites related to heptanal and (E,E)-2,4-nonadienal were screened, the most important of which were 6-Methylquinoline, L-Tyrosine, L-Asparagine, Adenosine, Valine (Table 1).

**TABLE 1:** Common DMs related to heptanal and (E,E)-2, 4-nonadienal in the blue module of BM tissue.

DMs	KEGG_pathway	Up/down	GS.(E,E)-2,4-nonadienal	GS.hexanal	GS.heptanal	MM.blue
L-tyrosine	Ubiquinone and other terpenoid-quinone biosynthesis; tyrosine metabolism; phenylalanine metabolism; phenylalanine, tyrosine and tryptophan biosynthesis; thiamine metabolism; aminoacyl-tRNA biosynthesis; metabolic pathways	Up	0.86679678	0.56659499	0.58551155	0.93150164
L-asparagine	Alanine, aspartate and glutamate metabolism; aminoacyl-tRNA biosynthesis; metabolic pathways; biosynthesis of amino acids	Up	0.82714735	0.56421369	0.55426162	0.89371582
Adenosine	Purine metabolism; metabolic pathways; neuroactive ligand-receptor interaction; vascular smooth muscle contraction	Up	0.82460886	0.78166194	0.75045521	0.84141729
L-isoleucine	Valine, leucine and isoleucine degradation; valine, leucine and isoleucine biosynthesis; aminoacyl-tRNA biosynthesis; metabolic pathways; 2-oxocarboxylic acid metabolism; biosynthesis of amino acids; ABC transporters	Up	0.78272462	0.53092618	0.58997034	0.83186834
L-pyroglutamic acid	Glutathione metabolism	Down	0.8461446	0.54609135	0.7028089	0.83481613
2-hydroxycinnamic acid	—	Up	0.88481871	0.58303684	0.6289965	0.95980337
DL-tryptophan	—	Up	0.86431014	0.69847007	0.69254193	0.94701208
Trans-3-indoleacrylic acid	—	Up	0.86401383	0.6968277	0.69022152	0.94698145
3-(3,4-dihydroxyphenyl)propanoic acid	—	Up	0.73358412	0.61866002	0.56325388	0.88109801
6-methylquinoline	—	Up	0.83491092	0.6731264	0.64709261	0.91940092
2-methyl-2,3,4,5-tetrahydro-1,5-benzoxazepin-4-one	—	Up	0.84232586	0.6129594	0.66681888	0.94715239
D-2-aminoadipic acid	—	Down	0.83396765	0.49296603	0.64701383	0.84458866
Valine	—	Up	0.84644358	0.63861214	0.75328642	0.9235164

## Discussion

In this study, WC chickens with a high-quality of meat scent were used. BM tissue was mainly composed of AAs (experimental group) and AF tissue was mainly composed of FAs (control group). We compared VOCs and metabolites in the two tissues using GC-MS and untargeted metabolomics to identify the precursors and metabolites of the main VOCs in chicken meat.

PCA is a dimension reduction technique that transforms multiple variables into several principal components (PCs) (Sharma et al., 2016). We can effectively use statistical data for quantitative analysis *via* PCA to reveal relationships between variables, identify the main components in complex relationships between objects, and understand the characteristics of components and their development rules. Thirty-six VOCs in BM tissue and 32 VOCs in AF tissue were detected, suggesting a greater diversity of VOCs in BM tissue. Previous studies showed that 32 and 27 VOCs were detected in BM tissue of Jingxing Yellow chicken and Tiannong Partridge chicken, and the differences from our results are likely due to different chicken breeds (Jin et al., 2021).

PCA was performed on VOCs in BM and AF tissue of 15 individuals. The results showed that samples had good repeatability and could be clearly distinguished between the two tissues, indicating that VOCs differed between tissues and might be derived from different precursors.

The hierarchical cluster diagram shows different accumulation patterns of VOCs that could be divided into three main groups. One group included heptacpsane, tetradecane, heneicosane, 6-methyl-octadecane, pentadecane, diethyl disulfide, octadecane, 4-ethyl -1-octy-3-ol, decyl ester-decanoic acid, and butylated hydroxytoluene. These metabolites were abundant in AF tissue, but not detected in BM tissue, hence we speculated that the precursors of these metabolites might be mostly from FAs. The second group included 1-octen-3-ol, (E,E)-2,4-nonadienal, benzaldehyde, 2,6,10-trimethyl-dodecane, 2-dodecenol, 1,9-nonanediol, 3-decyn-2-ol, (E)-2-nonen-1-ol, heptadecane, dodecanal, 2-hexyl-1-decanol, 3-butenyl-Oxirane. These VOCs were mainly found in BM tissue and not detected in AF tissue, hence we speculated that the precursors of these metabolites might be derived from AAs. The third group included heptanal, octanal, nonadecane, hexanal, (E)-2-octenal, hexadecanal and some other VOCs. The content pattern of these were similar in the two tissues, with some present at higher levels in BM or AF, but detected in both tissues, hence we speculated that the precursors of these compounds were derived from AAs or FAs or both.

PCA based on all VOCs revealed nine major VOCs across both tissues. Levels of nine representative VOCs in BM tissue were high, while levels of other four VOCs were low. Therefore, these nine VOCs are the main VOCs affecting BM tissue flavor, and the same is true for AF tissue. Hexanal, heptanal, octanal and nonanal were the most significant VOCs in both tissues, and hexanal appears to make the most significant contribution to flavor quality of broilers. Previous studies also showed that hexanal might be the main VOC affecting the flavor quality of broilers (Jin et al., 2021).

Comparison of the contents of these major VOCs in the two tissues showed that hexanal level in AF tissue was higher than that in BM. It has been reported that hexanal, pentaldehyde, nonanal, heptanal, (E)-octanal, (Z)-2-octanal, (E)-2-decanal, (E,E)-2-nonanal, and 2, 4-decdienal are formed by the oxidation or thermal oxidation decomposition of unsaturated FAs in food (Madruga et al., 2010; Sampaio et al., 2012). Thus, it can be inferred that FAs may be the main precursor substance of hexanal. Studies have shown that although hexanal is constantly oxidized in the heating process, hexanal and other aldehydes contribute greatly to the overall flavor of cooked beef, and excessive levels of hexanal can produce an unpleasant smell (Ma et al., 2012). Hexanal and heptanal are oxidation products of linoleic acid and arachidonic acid, respectively. The relative content of hexanal and nonanal can decrease the flavor and taste of ham (López-Pedrouso et al., 2019), consistent with our conclusion.

The flavor precursors that make up the basic tastes of cooked meat (sweet, salty, bitter, and sour) are non-volatile components of fresh meat (sugars, peptides, amino acids, inorganic salts, and organic acids) (Macleod, 1994). One study (Whitfield, 1992) showed that FAs such as 14:1, 16:1, 18:0, 18:1, 18:2 and 18:3 were related to beef flavor. It has also been shown that the flavor of species depends mainly on ketones, saturated aldehydes, FAs and unsaturated aldehydes, which play a major role in meat flavor (Melton, 1990). Among them, the AAs cysteine and methionine are considered to be the main contributors to the development of meat flavor (Werkhoff et al., 1990). In chicken stew, the taste activity values (TAV) of hexanal is between 20 and 160. Therefore, it plays a key role in the flavor perception of chicken soup (Qi et al., 2017). (E)-hept-2-enal-M, phenylacetaldehyde, and valeraldehyde are important modifiers of overall flavor in braised and fried chicken (Bi et al., 2021). Previous studies have shown that hexanal and 1-octen-3-ol are the main VOCs in Jingxing Yellow chicken, Tiannong Partridge chicken, and Wenchang chicken, confirming the main aroma VOCs in chicken, which provides a basis for breeding birds with improved meat flavor (Jin et al., 2021).

Benzaldehyde was mainly detected in BM tissue but not AF. It is speculated that benzaldehyde is mainly produced by AAs as precursors. Studies have shown that some aldehydes are derived from carbonyl groups produced by protein oxidation. For example, benzaldehyde is derived from the degradation product of phenylalanine through Strecker degradation. However, the sour taste of benzaldehyde can affect the aroma of yak (Huang et al., 2022), which further supports our conjecture. Furthermore, 1-octen-3-ol was also detected only in BM tissue, suggesting it may be derived from AAs. However, studies during soybean seed processing have shown that 1-octen-3-ol is formed from derivatives of unsaturated FAs (Matsui et al., 2018), which is inconsistent with our speculation. Moreover, since species differ, flavor substances may vary greatly from species to species after steaming (Yang et al., 2019; Wang et al., 2021). This is not consistent with our speculation, possibly because species differ, and flavor substances may vary significantly from species to species after steaming.

In a study on warmed-over flavors, chicken was described using the terms cardboard-like, chicken meat, rancid, vegetable oil, bread, toasted, and nut-like, among others (Byrne et al., 1999). Previous studies on the flavor characterization of meats show that chicken flavors can be mainly described by descriptors such as salty, fatty, brothy, and cardboardy (oxidized) (Byrne et al., 1999; Zhuang et al., 2007; Zhuang and Savage, 2008; Zhuang and Savage, 2011). The meat of the yellow-feather broiler has higher levels of nonvolatile (5′-nucleotides) and volatile compounds [(*E*)-2-octenal, (*E*)-2-nonenal, heptanol, and 2-decanone], a tougher texture, and it is chewier than meat from commercial white-feathered broiler breeds such as Arbor Acres and Avian (Tang et al., 2009; Long et al., 2015; Wang et al., 2015). Hexanal has a TAV ranging from 20 to 160 during the stewing process. Therefore, it played a key role in the perception of flavor in chicken soup (Qi et al., 2017).

Previous studies have focused on of identification VOCs (Fan et al., 2018; Xu et al., 2020; Bi et al., 2021; Jin et al., 2021). However, the intermediate metabolites of VOCs remain unclear. The origin of different VOCs can be further determined by untargeted metabolite analysis between different tissues. PCA and hierarchical clustering methods detected similarities and differences between BM and AF tissue samples. There was good repeatability between the replicate samples in each treatment group, and significant differences between BM and AF tissues. PLS-DA results also tend to be consistent, confirming the reliability of the metabolome data.

To explore the precursors of VOCs from the perspective of metabolites, we performed WGCNA of nine major VOCs in BM tissue among all metabolites identified by metabolome analysis. In BM tissue, the blue module was identified to related with three VOCs, while it was screened for phenotypically relevant key metabolites based on GS and MM values. Twenty-one common DMs were obtained by analyzing the differences in metabolites related to three VOCs: hexanal, heptanal, and (E,E)-2,4-nonadienal.

This analysis method can identify important metabolites regulating the three traits, which is more conducive to improving of high-quality broiler breeding. It has been reported that linoleic acid is a prerequisite for the formation of hexanal (Morita and Tokita, 2006; Morita and Tokita, 2008). Since hexanal was detected at high levels in BM and AF tissues, we hypothesized that FAs and some AAs may be involved in the synthesis of hexanal. The common DMs significantly related to (E, E)-2,4-nonadienal and heptanal were enriched in AA metabolism pathways (such as Ubiquinone and other terpenoid-quinone biosynthesis, Tyrosine metabolism, Phenylalanine metabolism, Phenylalanine-tyrosine and tryptophan biosynthesis, Glutathione metabolism). Meanwhile, (E, E)-2,4-nonadienal was only detected in BM tissue, while heptanal was significantly more abundant in BM than AF. Therefore, we inferred that AAs played a vital role in forming (E, E)-2,4-nonadienal, and heptanal. Studies have shown that the main VOCs identified in cooked meat pies are hydrocarbons, aldehydes, ketones, alcohols and sulfur compounds produced by the degradation of FAs and AAs (Huan et al., 2005), consistent with our results.

The results illuminate the precursors of the main VOCs, revealing details of nutritional regulation of broilers. However, we tested WC chickens, which have high fat content and superior flavor quality, hence they are not representative of all broiler breeds. Therefore, the experimental results can also be repeated using other broiler breeds, and if consistent results could be obtained would be more convincing. Secondly, considering the restrictive number of individuals, a larger sample size could be selected for subsequent experiments to obtain more credible results. Moreover, we will also validate the results in terms of experiments to ensure more credibility.

## Conclusion

In summary, this study identified the precursors of some key volatile compounds in chicken meat. It confirmed that AAs are the main precursors of 1-octen-3-ol, (E,E)-2,4-nonadienal, heptanal and other compounds while FAs are main precursors of diethyl disulfide. Nevertheless, hexanal may be formed from a combination of AAs and a small number of FAs as precursors. The results provide a basis for understanding the volatile compounds in chicken meat. Furthermore, the findings will assist the design more rational strategies for improving the flavor quality of broilers under subsequent nutritional conditions.

## Data Availability

The original contributions presented in the study are included in the article/Supplementary Materials, further inquiries can be directed to the corresponding author.

## Abbreviations

AAs, Amino Acids; AF, Abdominal Fat; BM, Breast Muscle; DFA, Discriminant Factor Analysis; DMs, Differential Metabolites; FAs, Fatty acids; FC, Fold Change; GC-MS, Gas Chromatography-Mass Spectrometry; GS, Gene Significance; KEGG, Kyoto Encyclopedia of Genes and Genomes; MM, Module Membership; PC1, First Principal Component; PCA, Principal Component Analysis; PLS-DA, Partial Least Squares Discriminant Analysis; PUFAs, Polyunsaturated FAs; QC, Quality Control; SEM, Mean ± Standard Error of the Mean; SPME, Solid Phase Microextraction; TAV, Taste activity values; TOM, Topology Overlap Matrix; VIP, Variable Importance in the Projection; VOCs, Volatile Organic Compounds; WC, Wenchang; WGCNA, Weighted Gene Co-expression Network Analysis.
